# Supplementary figures and images for: Plastid phylogenomics of Pleurothallidinae (Orchidaceae): Conservative plastomes, new variable markers, and comparative analyses of plastid, nuclear, and mitochondrial data
Source: PLoS One. 2021 Aug 27;16(8):e0256126. doi: 10.1371/journal.pone.0256126 (PMC8396723; doi:10.1371/journal.pone.0256126)

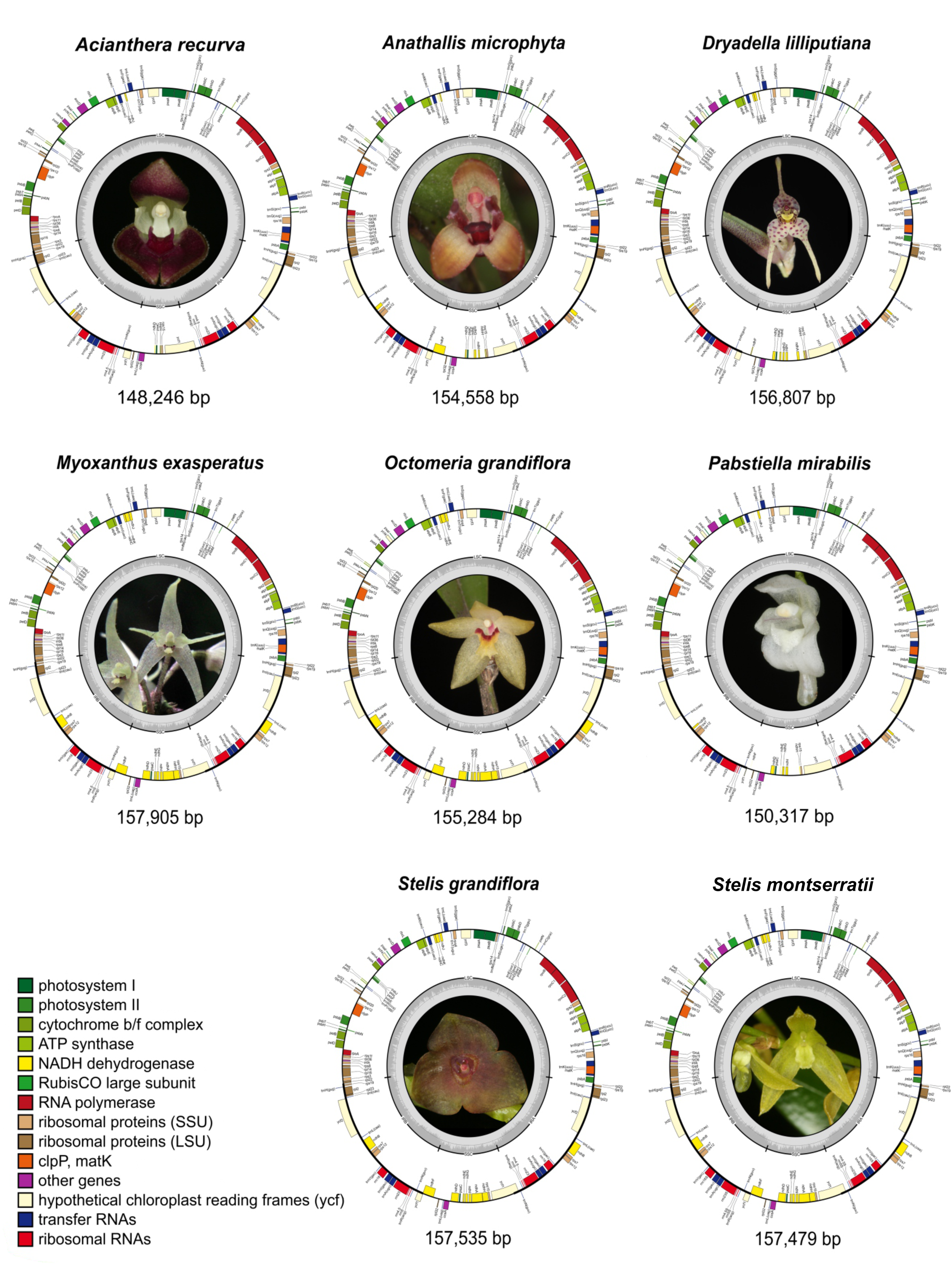

Supplement: S1 Fig — Genes are represented by rectangles, for which functions are identified by colors as shown in the legend. Genes placed inside the circle are transcribed clockwise, and those outside the circle are transcribed counter-clockwise. The gray inner circle is the GC content graph. Images of the sequenced species were taken by Eric C. Smidt except Marcelo Rodrigues for Myoxanthus exasperatus. (TIF) [file pone.0256126.s001.tif]

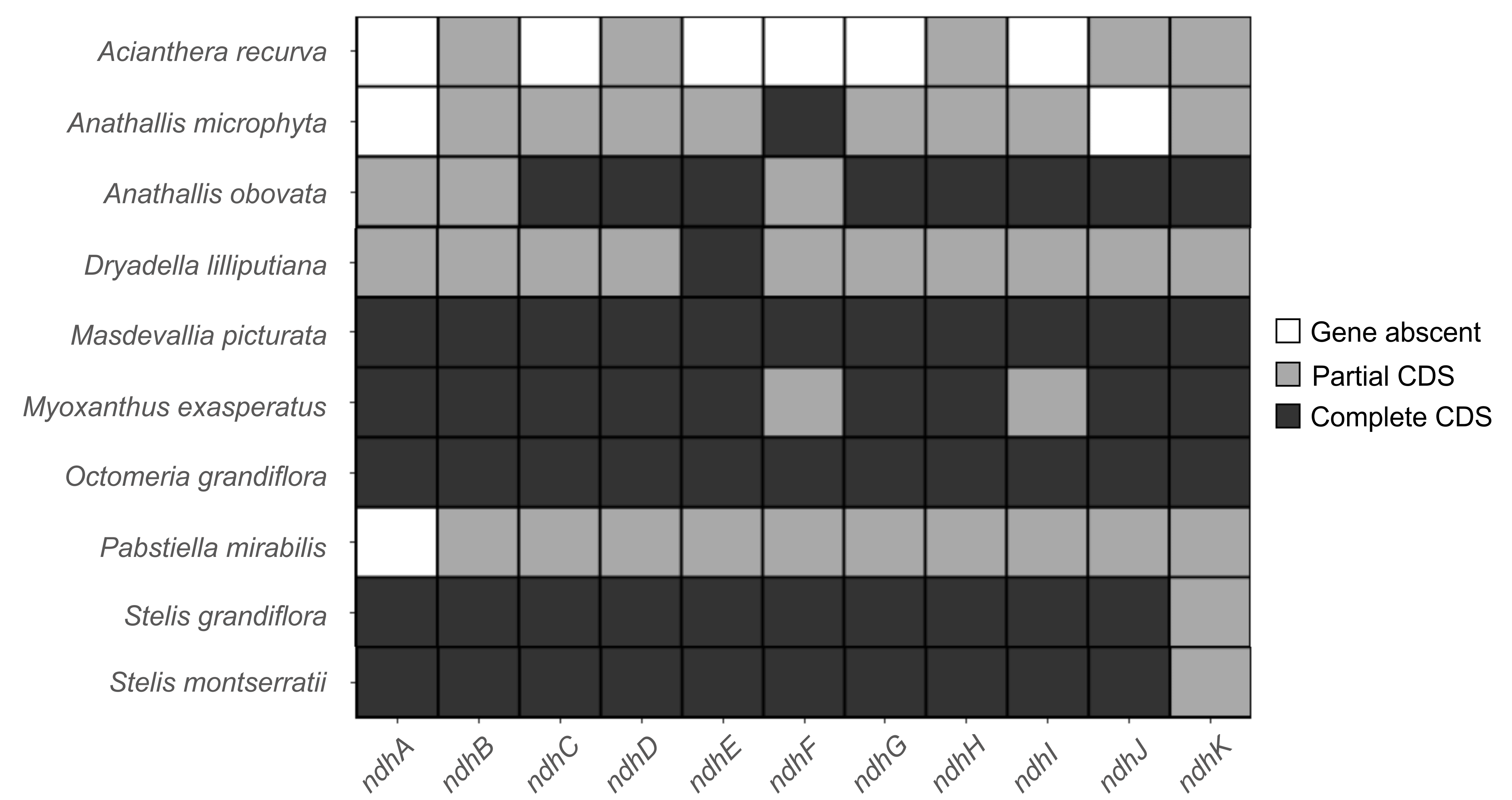

Supplement: S2 Fig — White squares = complete gene losses, light-grey squares = truncated reading frames (pseudogenes), and dark-grey squares = complete CDSs. (TIF) [file pone.0256126.s002.tif]

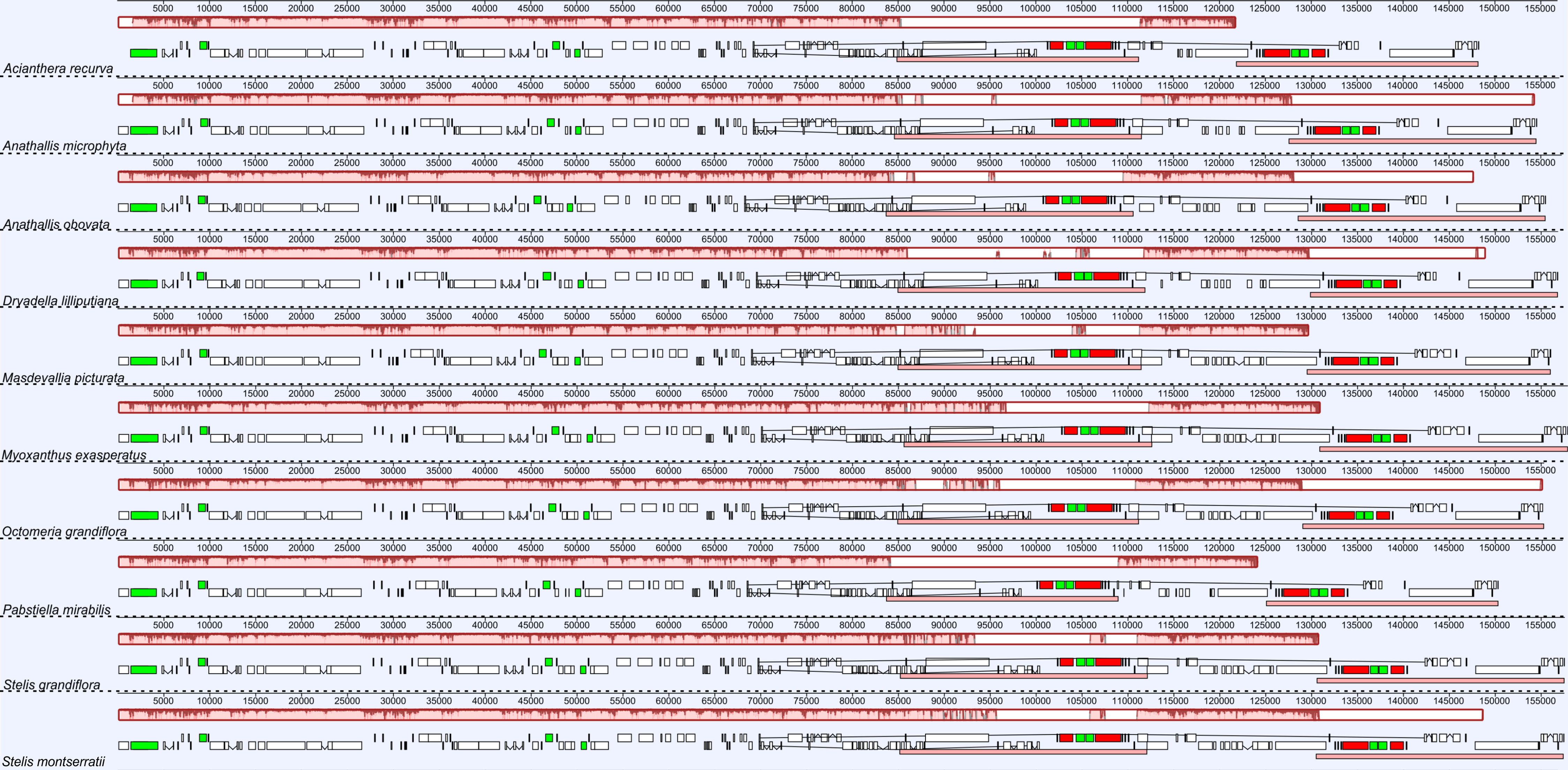

Supplement: S3 Fig — (TIF) [file pone.0256126.s003.tif]

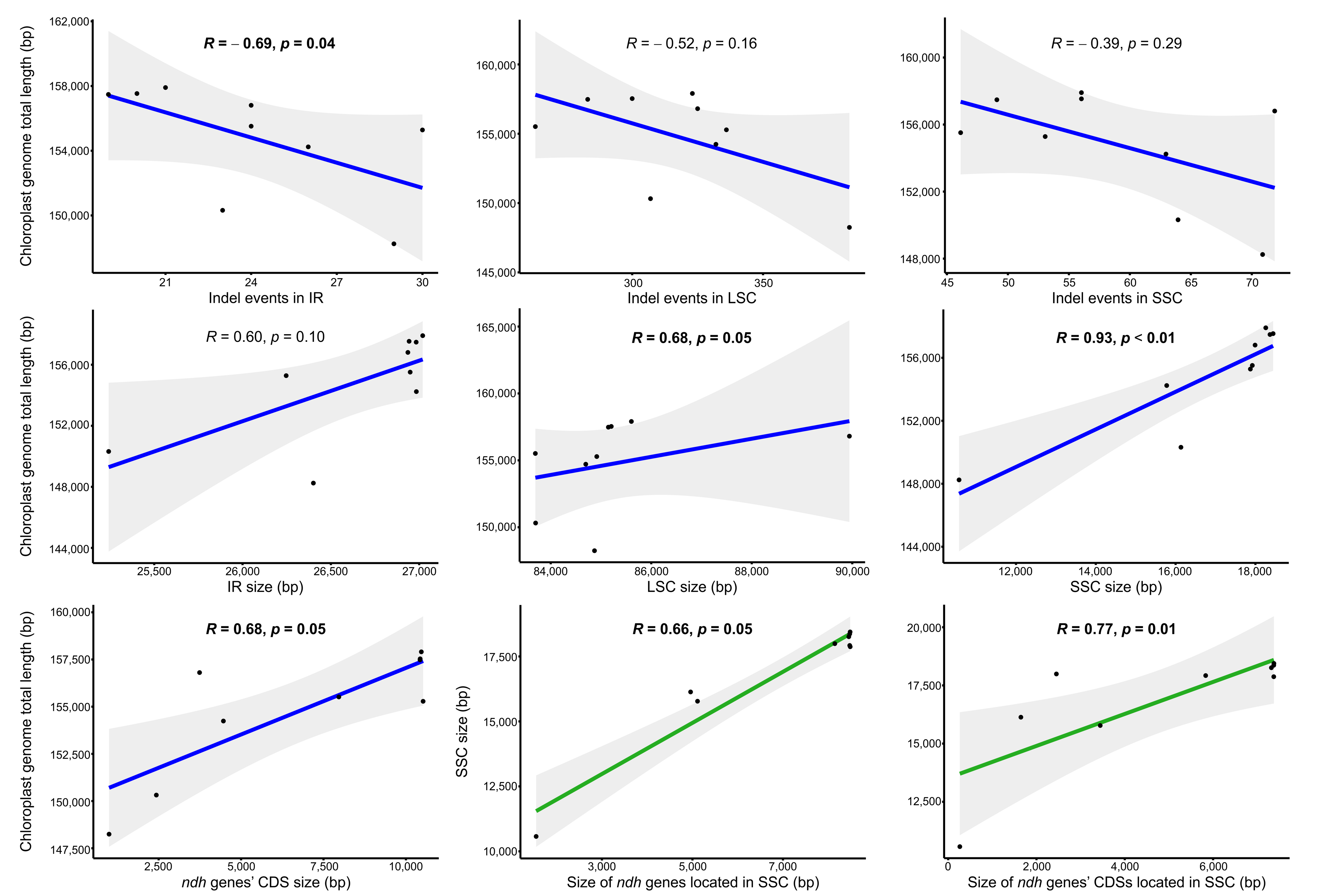

Supplement: S4 Fig — (TIF) [file pone.0256126.s004.tif]

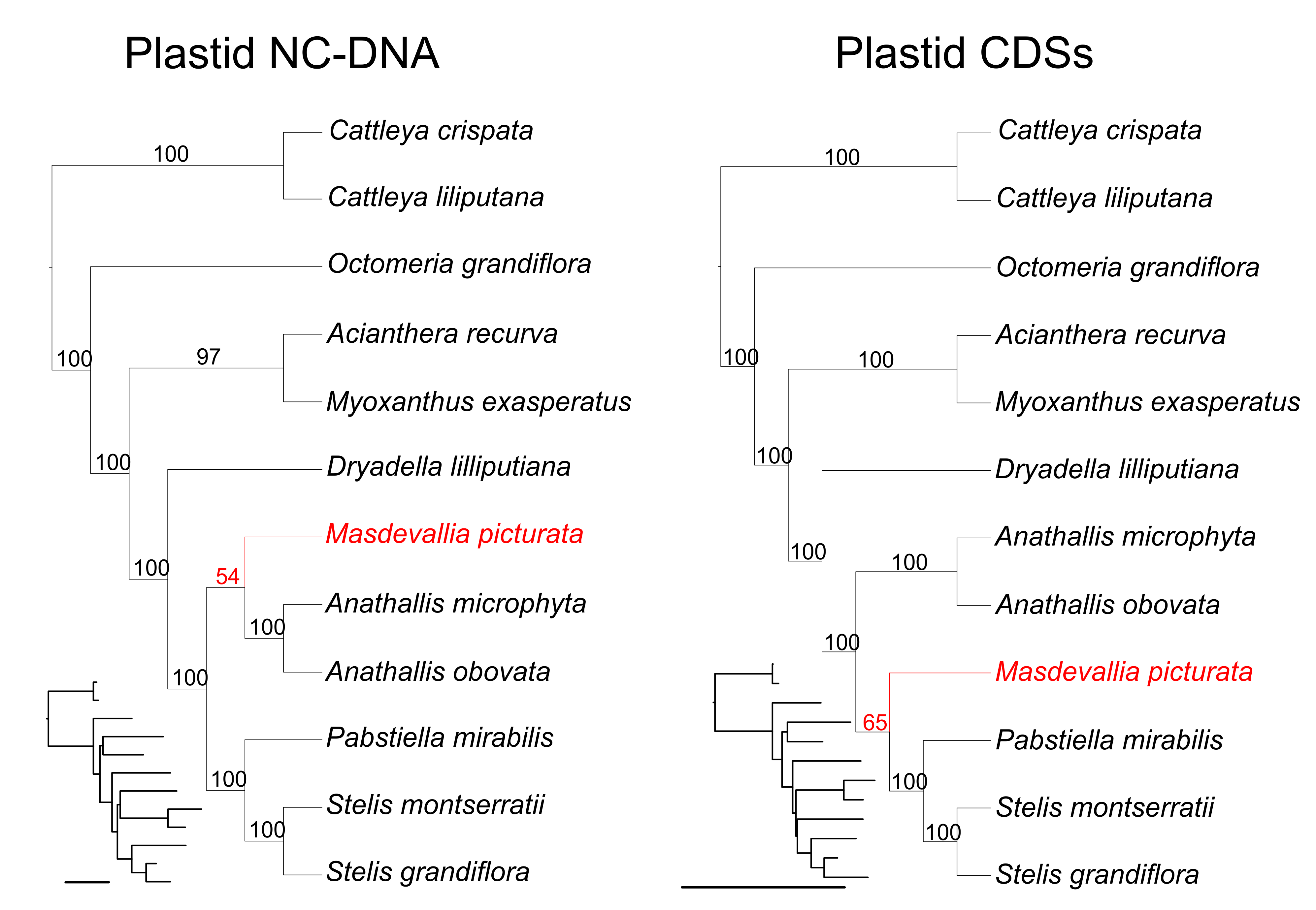

Supplement: S5 Fig — Numbers on branches are the bootstrap percentages; a tree with proportional branch lengths is on the left of each tree, which bars represent 0.02 nucleotide substitutions per site. (TIF) [file pone.0256126.s005.tif]

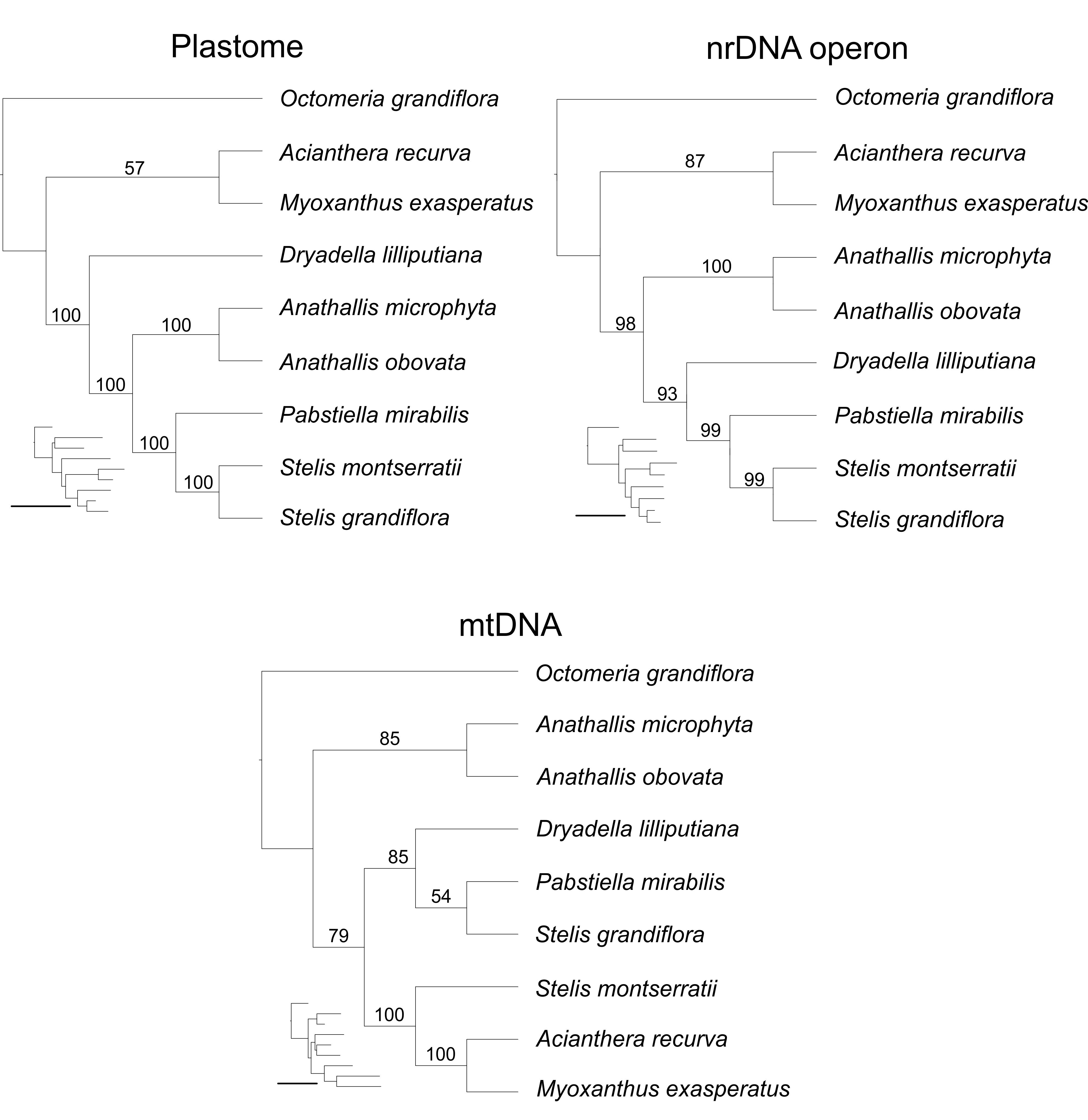

Supplement: S6 Fig — Numbers on branches refer to bootstrap percentages; a tree with proportional branch lengths is on the left of each tree, which bars represent 0.02 nucleotide substitutions per site. (TIF) [file pone.0256126.s006.tif]
