# Supplementary material for: Plastid phylogenomics of Pleurothallidinae (Orchidaceae): Conservative plastomes, new variable markers, and comparative analyses of plastid, nuclear, and mitochondrial data
Source: PLoS One. 2021 Aug 27;16(8):e0256126. doi: 10.1371/journal.pone.0256126 (PMC8396723; doi:10.1371/journal.pone.0256126)
Supplement: S1 Table — Accessions in boldface are sequences generated in this study. All vouchers provided are deposited at UPCB herbarium. (PDF) [file pone.0256126.s007.pdf]

**Subtribe**	**Affinity**	**Taxon**	**Voucher**	**GenBank accession (plastome)**	**GenBank accession (nrDNA)**	**GenBank accession (mtDNA)**
Laeliinae	-	*Cattleya crispata* (Thunb.) Van den Berg	unvouchered	KP168671	-	-
Laeliinae	-	*Cattleya liliputana* (Pabst) Van den Berg	unvouchered	KP202881	-	-
Pleurothallidinae	*Acianthera*	*Acianthera recurva* (Lindl.) Pridgeon & M.W. Chase	D.C. Imig 500 (UPCB)	**MW375123**	**MW364931**	**MW562896–MW562941**
Pleurothallidinae	*Lepanthes*	*Anathallis microphyta* (Barb. Rodr.) C.O.Azevedo & Van den Berg	M.C. Santos 25 (UPCB)	**MW375124**	**MW364932**	**MW562942–MW563001**
Pleurothallidinae	*Lepanthes*	*Anathallis obovata* (Lindl.) Pridgeon & M.W. Chase	M.C. Santos 22 (UPCB)	MH979332	**MW364933**	**MW563002–MW563053**
Pleurothallidinae	*Masdevallia*	*Masdevallia picturata* Rchb. f.	unvouchered	KJ566305	-	-
Pleurothallidinae	*Octomeria*	*Octomeria grandiflora* Lindl.	D.C. Imig 503 (UPCB)	**MW375128**	**MW364937**	**MW563178–MW563221**
Pleurothallidinae	*Pleurothallis*	*Pabstiella mirabilis* (Schltr.) Brieger & Senghas	A.L.V. Toscano de Brito 3287 (UPCB)	**MW375130**	**MW364938**	**MW563222–MW563270**
Pleurothallidinae	*Pleurothallis*	*Stelis grandiflora* Lindl.	M.E. Engels 1600 (UPCB)	**MW375129**	**MW364939**	**MW563271–MW563320**
Pleurothallidinae	*Pleurothallis*	*Stelis montserratii* (Porsch) Karremans	D.C. Imig 410 (UPCB)	**MW375125**	**MW364934**	**MW563054–MW563091**
Pleurothallidinae	*Restrepia*	*Myoxanthus exasperatus* (Lindl.) Luer	T.F. Santos 326 (UPCB)	**MW375127**	**MW364936**	**MW563137–MW563177**
Pleurothallidinae	*Specklinia*	*Dryadella lilliputiana* (Cogn.) Luer	D.C. Imig 469 (UPCB)	**MW375126**	**MW364935**	**MW563092–MW563136**
