# Supplementary material for: Plastid phylogenomics of Pleurothallidinae (Orchidaceae): Conservative plastomes, new variable markers, and comparative analyses of plastid, nuclear, and mitochondrial data
Source: PLoS One. 2021 Aug 27;16(8):e0256126. doi: 10.1371/journal.pone.0256126 (PMC8396723; doi:10.1371/journal.pone.0256126)
Supplement: S2 Table — *See Mauad et al. [30]. (PDF) [file pone.0256126.s008.pdf]

| Taxon | # raw reads | Raw reads mean length (bp) | # post-trim reads | Post-trim reads mean length (bp) | # reads mapped to plastomes | Plastome mean coverage (x) | # raw reads mapped to mitochondrion | mtDNA mean coverage (x) | # raw reads mapped to nrDNA operon | nrDNA operon mean coverage (x) |
|---|---|---|---|---|---|---|---|---|---|---|
| ***Acianthera recurva*** | 614,726 | 179.22 | 604,109 | 179.38 | 57,687 (09.56%) | 68.3 | 34,916 (05.68%) | 112.1 | 976 (00.16%) | 23.9 |
| ***Anathallis microphyta*** | 1,331,958 | 127.44 | 1,221,040 | 130.71 | 266,857 (22.72%) | 195.3 | 110,533 (08.30%) | 35.5 | 4,472 (00.33%) | 65.1 |
| ***Anathallis obovata*** | 1,378,972 | * | * | * | * | * | 75,501 (05.47%) | 30.5 | 3,207 (00.23%) | 59.6 |
| ***Dryadella lilliputiana*** | 1,284,838 | 159.42 | 1,239,756 | 161.73 | 230,188 (18.58%) | 241.2 | 112,230 (08.73%) | 62.4 | 4,647 (00.36%) | 95.4 |
| ***Myoxanthus exasperatus*** | 898,306 | 130.75 | 839,586 | 134.70 | 189,429 (22.64%) | 163.7 | 92,521 (10.30%) | 71.2 | 3,745 (00.41%) | 58.9 |
| ***Octomeria grandiflora*** | 1,142,910 | 167.76 | 1,095,295 | 171.23 | 121,474 (11.11%) | 133.5 | 53,065 (04.64%) | 29.0 | 2,630 (00.23%) | 52.7 |
| ***Pabstiella mirabilis*** | 1,231,612 | 171.72 | 1,195,781 | 172.83 | 60,445 (05.10%) | 182.9 | 40,170 (03.26%) | 24.0 | 6,036 (00.49%) | 135.4 |
| ***Stelis grandiflora*** | 1,120,894 | 138.39 | 1,046,794 | 143.58 | 150,757 (14.42%) | 135.7 | 62,856 (05.61%) | 27.0 | 2,689 (00.24%) | 47.7 |
| ***Stelis montserratii*** | 826,542 | 188.57 | 816,984 | 187.57 | 116,542 (14.29%) | 131.8 | 56,799 (06.87%) | 57.8 | 683 (00.08%) | 16.6 |
