# Supplementary material for: Plastid phylogenomics of Pleurothallidinae (Orchidaceae): Conservative plastomes, new variable markers, and comparative analyses of plastid, nuclear, and mitochondrial data
Source: PLoS One. 2021 Aug 27;16(8):e0256126. doi: 10.1371/journal.pone.0256126 (PMC8396723; doi:10.1371/journal.pone.0256126)
Supplement: S3 Table — *Genes with introns. (PDF) [file pone.0256126.s009.pdf]

Gene groups	Gene names
**Ribosomal RNA**	*5S, 18S, 26S*
**Transport RNA**	*trnE* –UUC, *trnK* –UUU, *trnL* –UAA, *trnM* –CAU, *trnQ* –UUG, *trnR* –GCG, *trnY* –AUA, *trnY* –GUA
**Ribosome small subunit**	*rps12*
**Maturase**	*matR*
**Apocytochrome b**	*cob*
**Cytochrome c biosynthesis**	*ccmB, ccmC, ccmFc*, ccmFN1, ccmFN2*
**Cytochrome c oxidase subunits**	*cox1, cox2*, cox3*
**ATP synthase subunits**	*atp1, atp4, atp6, atp8, atp9*
**NADH dehydrogenase subunits**	*nad1*, nad2*, nad3, nad4*, nad4L, nad5*, nad6, nad7*, nad9*
**Transport membrane protein**	*mttb*
