# Supplementary material for: Plastid phylogenomics of Pleurothallidinae (Orchidaceae): Conservative plastomes, new variable markers, and comparative analyses of plastid, nuclear, and mitochondrial data
Source: PLoS One. 2021 Aug 27;16(8):e0256126. doi: 10.1371/journal.pone.0256126 (PMC8396723; doi:10.1371/journal.pone.0256126)
Supplement: S4 Table — aGenes with introns, bduplicated genes (in IRs), cpartially duplicated genes, *pseudogenes. (PDF) [file pone.0256126.s010.pdf]

**Gene groups**	**Gene names**
**Ribosomal RNA**	*rrn4.5*[b]; *rrn5*[b]; *rrn16*[b]; *rrn23*[b]
**Transport RNA**	*trnA* –UGC[ab]; *trnC* –GCA; *trnD* –GUC; *trnE* –UUC; *trnF* –GAA; *trnfM* –CAU; *trnG* –GCC; *trnG* –UCC[a]; *trnH* –GUG[b]; *trnI* –CAU[b]; *trnI* –GAU[ab]; *trnK* –UUU[a]; *trnL* –CAA[b]; *trnL* –UAA[a]; *trnL* –UAG; *trnM* –CAU; *trnN* –GUU[b]; *trnP* –UGG; *trnQ* –UUG; *trnR* –ACG[b]; *trnR* –UCU; *trnS* –GCU; *trnS* –GGA; *trnS* –UGA; *trnT* –GGU; *trnT* –UGU; *trnV* –GAC[b]; *trnV* –UAC[a]; *trnW* –CCA; *trnY* –GUA
**Ribosome small subunit**	*rps2*; *rps3*; *rps4*; *rps7*[b]; *rps8*; *rps11*; *rps12*[ab]; *rps14*; *rps15*; *rps16*[a]; *rps18*; *rps19*[b]
**Ribosome large subunit**	*rpl2*[ab]; *rpl14*; *rpl16*[a]; *rpl20*; *rpl22*; *rpl23*[b]; *rpl32*; *rpl33*; *rpl36*
**RNA-polimerase**	*rpoA*; *rpoB*; *rpoC1*[a]; *rpoC2*
**Translation initiation factor**	*infA*
**Maturase**	*matK*
**Photosystem I (PSI) subunits**	*psaA*; *psaB*; *psaC*; *psaI*; *psaJ*; *ycf3*[a]; *ycf4*
**Photosystem II (PSII) subunits**	*psbA*; *psbB*; *psbC*; *psbD*; *psbE*; *psbF*; *psbH*; *psbI*; *psbJ*; *psbK*; *psbL*; *psbM*; *psbN*; *psbT*; *psbZ*
**Cytochrome b_6f subunits**	*petA*; *petB*[a]; *petD*[a]; *petG*; *petL*; *petN*
**ATP synthase subunits**	*atpA*; *atpB*; *atpE*; *atpF*; *atpH*; *atpI*
**NADH dehydrogenase subunits***	*ndhA*[a]; *ndhB*[ab]; *ndhC*; *ndhD*; *ndhE*; *ndhF*; *ndhG*; *ndhH*; *ndhI*; *ndhJ*; *ndhK*
**Rubisco**	*rbcL*
**Chloroplast envelope membrane protein**	*cemA*
**Acetyl-CoA carboxylase beta subunit**	*accD*
**Cytochrome c biogenesis protein**	*ccsA*
**Clp protease subunit**	*clpP*[a]
**TIC complex component**	*ycf1*[c]
**Unknown function**	*ycf2*[b]
