# Supplementary material for: Plastid phylogenomics of Pleurothallidinae (Orchidaceae): Conservative plastomes, new variable markers, and comparative analyses of plastid, nuclear, and mitochondrial data
Source: PLoS One. 2021 Aug 27;16(8):e0256126. doi: 10.1371/journal.pone.0256126 (PMC8396723; doi:10.1371/journal.pone.0256126)
Supplement: S5 Table — The plastome of Madevallia picturata was the reference for all indels. (PDF) [file pone.0256126.s011.pdf]

**Dataset**	**IR**	**LSC**	**SSC**	**Total**
**Pleurothallidinae**	134	1,899	384	2,417
Acianthera recurva	29	383	71	483
Anathallis microphyta	26	330	62	418
Anathallis obovata	24	263	46	333
Dryadella lilliputiana	24	325	72	421
Myoxanthus exasperatus	21	323	56	400
Octomeria grandiflora	30	336	53	419
Pabstiella mirabilis	23	307	64	394
Stelis grandiflora	20	300	56	376
Stelis montserratii	19	283	49	351
