# Supplementary material for: Plastid phylogenomics of Pleurothallidinae (Orchidaceae): Conservative plastomes, new variable markers, and comparative analyses of plastid, nuclear, and mitochondrial data
Source: PLoS One. 2021 Aug 27;16(8):e0256126. doi: 10.1371/journal.pone.0256126 (PMC8396723; doi:10.1371/journal.pone.0256126)
Supplement: S6 Table — Species names were abbreviated as follows: ACIRE = Acianthera recurva, ANAMI = Anathallis microphyta, ANAOB = A. obovata, DRYLI = Dryadella lilliputiana, MASPI = Masdevallia infracta, MYOEX = Myoxanthus exasperatus, OCTGR = Octomeria grandiflora, PABMI = Pabstiella mirabilis, STEGR = Stelis grandiflora, and STEMO = Stelis montserratii. (PDF) [file pone.0256126.s012.pdf]

AA	Codon	ACIRE		ANAMI		ANAOB		DRYLI		MASPI		MYOEX		OCTGR		PABMI		STEGR		STEMO	
		RF	RSCU	RF	RSCU	RF	RSCU	RF	RSCU	RF	RF	RSCU	RSCU	RF	RSCU	RF	RSCU	RF	RSCU	RF	RSCU
Ala	GCA	1.63	1.21	1.61	1.19	1.65	1.20	1.59	1.19	1.65	1.64	1.22	1.22	1.64	1.22	1.61	1.21	1.65	1.22	1.63	1.21
	GCC	0.73	0.54	0.73	0.54	0.76	0.55	0.74	0.56	0.75	0.75	0.56	0.55	0.75	0.56	0.75	0.56	0.76	0.56	0.76	0.57
	GCG	0.49	0.37	0.50	0.37	0.50	0.36	0.54	0.40	0.49	0.47	0.35	0.36	0.47	0.35	0.47	0.35	0.50	0.37	0.48	0.35
	GCT	2.51	1.87	2.57	1.90	2.58	1.88	2.48	1.85	2.51	2.49	1.86	1.86	2.50	1.86	2.50	1.87	2.50	1.84	2.52	1.86
Arg	AGA	1.97	1.90	1.93	1.92	1.92	1.89	1.95	1.90	1.86	1.88	1.91	1.91	1.91	1.92	1.94	1.95	2.01	1.95	1.89	1.93
	AGG	0.64	0.62	0.59	0.59	0.62	0.61	0.63	0.62	0.57	0.58	0.59	0.59	0.58	0.59	0.59	0.59	0.59	0.58	0.58	0.59
	CGA	1.42	1.37	1.37	1.37	1.38	1.36	1.42	1.38	1.34	1.34	1.36	1.37	1.34	1.35	1.34	1.35	1.39	1.36	1.32	1.34
	CGC	0.36	0.35	0.33	0.33	0.34	0.34	0.32	0.32	0.34	0.35	0.35	0.35	0.33	0.33	0.31	0.32	0.34	0.33	0.34	0.35
	CGG	0.39	0.37	0.40	0.40	0.40	0.39	0.40	0.39	0.37	0.37	0.37	0.38	0.39	0.40	0.38	0.38	0.38	0.37	0.38	0.39
	CGT	1.44	1.39	1.39	1.38	1.41	1.39	1.41	1.37	1.36	1.38	1.40	1.39	1.39	1.40	1.39	1.40	1.44	1.40	1.37	1.40
Asn	AAC	1.01	0.42	0.93	0.39	0.92	0.41	0.98	0.41	0.92	0.91	0.39	0.40	0.92	0.40	0.93	0.39	0.94	0.40	0.90	0.39
	AAT	3.77	1.58	3.79	1.60	3.60	1.59	3.76	1.59	3.73	3.73	1.60	1.60	3.71	1.60	3.77	1.60	3.76	1.60	3.72	1.61
Asp	GAC	0.71	0.34	0.76	0.38	0.69	0.35	0.73	0.36	0.71	0.72	0.36	0.35	0.72	0.36	0.70	0.35	0.72	0.35	0.71	0.36
	GAT	3.40	1.65	3.27	1.62	3.30	1.65	3.30	1.63	3.29	3.26	1.64	1.64	3.27	1.64	3.27	1.65	3.36	1.64	3.26	1.64
Cys	TGC	0.27	0.47	0.25	0.43	0.24	0.41	0.25	0.44	0.24	0.24	0.41	0.41	0.24	0.41	0.25	0.43	0.28	0.48	0.25	0.43
	TGT	0.87	1.53	0.90	1.57	0.92	1.58	0.90	1.56	0.92	0.93	1.59	1.58	0.93	1.58	0.93	1.57	0.90	1.52	0.91	1.57
Gln	CAA	2.89	1.55	2.77	1.53	2.80	1.54	2.87	1.55	2.72	2.75	1.54	1.53	2.74	1.53	2.70	1.53	2.83	1.53	2.73	1.54
	CAG	0.84	0.45	0.84	0.46	0.83	0.46	0.83	0.45	0.83	0.81	0.46	0.47	0.83	0.47	0.82	0.47	0.87	0.47	0.81	0.45
Glu	GAA	4.30	1.51	4.09	1.50	4.14	1.52	4.24	1.52	4.08	4.07	1.53	1.53	4.04	1.51	4.10	1.51	4.20	1.52	4.05	1.52
	GAG	1.37	0.48	1.34	0.49	1.28	0.47	1.32	0.47	1.26	1.26	0.47	0.47	1.31	0.49	1.32	0.49	1.33	0.48	1.27	0.48
Gly	GGA	2.62	1.57	2.68	1.58	2.72	1.60	2.63	1.57	2.74	2.77	1.62	1.61	2.76	1.62	2.76	1.63	2.64	1.57	2.76	1.62
	GGC	0.68	0.40	0.68	0.40	0.68	0.40	0.66	0.39	0.65	0.64	0.37	0.38	0.65	0.38	0.63	0.37	0.69	0.41	0.66	0.39
	GGG	1.13	0.68	1.15	0.67	1.13	0.67	1.16	0.69	1.14	1.15	0.67	0.67	1.12	0.66	1.15	0.68	1.16	0.69	1.14	0.67
	GGT	2.25	1.34	2.28	1.34	2.27	1.33	2.23	1.33	2.24	2.26	1.32	1.32	2.26	1.33	2.21	1.31	2.24	1.33	2.24	1.32
His	CAC	0.54	0.43	0.55	0.44	0.54	0.44	0.53	0.43	0.56	0.54	0.45	0.47	0.54	0.46	0.53	0.45	0.54	0.43	0.54	0.45
	CAT	1.98	1.57	1.93	1.55	1.92	1.56	1.94	1.57	1.80	1.83	1.54	1.52	1.82	1.54	1.81	1.54	1.97	1.57	1.85	1.54
Ile	ATA	2.56	0.92	2.64	0.93	2.67	0.94	2.53	0.90	2.70	2.72	0.94	0.93	2.70	0.94	2.71	0.94	2.46	0.89	2.72	0.94
	ATC	1.64	0.59	1.68	0.59	1.67	0.59	1.66	0.59	1.69	1.67	0.58	0.58	1.73	0.60	1.69	0.59	1.69	0.61	1.68	0.58
	ATT	4.12	1.48	4.15	1.47	4.17	1.47	4.18	1.50	4.27	4.25	1.47	1.48	4.17	1.45	4.23	1.47	4.13	1.50	4.22	1.47
Leu	CTA	1.45	0.85	1.38	0.80	1.41	0.81	1.41	0.81	1.44	1.44	0.82	0.82	1.42	0.81	1.44	0.82	1.38	0.81	1.47	0.84
	CTC	0.61	0.36	0.65	0.38	0.66	0.38	0.65	0.37	0.66	0.64	0.37	0.38	0.67	0.38	0.68	0.39	0.62	0.36	0.65	0.37
	CTG	0.68	0.40	0.67	0.39	0.68	0.39	0.69	0.40	0.71	0.67	0.38	0.40	0.67	0.39	0.66	0.37	0.72	0.42	0.69	0.40
	CTT	2.09	1.24	2.16	1.25	2.15	1.24	2.25	1.30	2.18	2.23	1.27	1.25	2.23	1.27	2.23	1.28	2.11	1.24	2.23	1.27
	TTA	3.19	1.89	3.36	1.96	3.40	1.96	3.34	1.93	3.47	3.42	1.95	1.98	3.41	1.95	3.43	1.96	3.28	1.92	3.44	1.96
	TTG	2.11	1.25	2.07	1.21	2.08	1.20	2.04	1.18	2.03	2.08	1.19	1.16	2.08	1.19	2.06	1.17	2.09	1.23	2.06	1.17

**Lys**	**AAA**	4.38	1.52	4.20	1.49	4.12	1.50	4.10	1.50	4.22	1.49	4.19	1.51	4.06	1.51	4.15	1.51	4.30	1.50	4.06	1.48
	**AAG**	1.39	0.48	1.41	0.50	1.38	0.50	1.36	0.50	1.42	0.50	1.34	0.48	1.32	0.49	1.34	0.49	1.42	0.50	1.42	0.52
**Met**	**ATG**	2.31	1.00	2.37	1.00	2.44	1.00	2.46	1.00	2.41	1.00	2.45	1.00	2.46	1.00	2.47	1.00	2.38	1.00	2.45	1.00
**Phe**	**TTC**	1.96	0.70	2.09	0.72	2.01	0.70	2.01	0.69	1.99	0.70	1.97	0.68	2.01	0.69	2.03	0.70	1.99	0.70	2.01	0.69
	**TTT**	3.64	1.30	3.73	1.28	3.70	1.29	3.81	1.31	0.36	1.29	3.82	1.32	3.78	1.30	3.78	1.30	3.64	1.29	3.79	1.30
**Pro**	**CCA**	1.08	1.08	1.09	1.09	1.14	1.13	1.15	1.15	1.09	1.09	1.17	1.17	1.15	1.15	1.15	1.15	1.09	1.10	1.15	1.14
**Pro**	**CCC**	0.90	0.90	0.88	0.89	0.87	0.86	0.87	0.87	0.91	0.91	0.85	0.84	0.88	0.87	0.86	0.86	0.88	0.89	0.87	0.86
	**CCG**	0.44	0.44	0.41	0.41	0.43	0.42	0.41	0.41	0.45	0.45	0.41	0.41	0.42	0.42	0.42	0.42	0.44	0.44	0.41	0.41
	**CCT**	1.57	1.57	1.58	1.59	1.60	1.58	1.56	1.56	1.54	1.54	1.58	1.57	1.56	1.55	1.58	1.57	1.53	1.55	1.60	1.57
**Ser**	**AGC**	0.41	0.33	0.41	0.32	0.39	0.32	0.39	0.30	0.41	0.32	0.40	0.32	0.39	0.30	0.39	0.31	0.40	0.31	0.39	0.30
	**AGT**	1.61	1.29	1.61	1.27	1.47	1.19	1.63	1.27	1.59	1.26	1.61	1.26	1.64	1.28	1.62	1.27	1.61	1.27	1.64	1.28
	**TCA**	1.47	1.18	1.50	1.18	1.46	1.19	1.57	1.22	1.51	1.19	1.58	1.24	1.53	1.19	1.55	1.22	1.48	1.17	1.57	1.22
	**TCC**	1.12	0.90	1.17	0.92	1.22	0.99	1.15	0.90	1.14	0.90	1.12	0.87	1.15	0.90	1.15	0.90	1.19	0.94	1.14	0.89
	**TCG**	0.59	0.47	0.58	0.46	0.60	0.49	0.61	0.47	0.62	0.49	0.60	0.47	0.64	0.50	0.62	0.49	0.59	0.47	0.59	0.46
	**TCT**	2.27	1.82	2.33	1.84	2.24	1.82	2.32	1.81	2.32	1.83	2.33	1.83	2.34	1.82	2.32	1.81	2.33	1.83	2.34	1.83
**Stop**	**TAA**	0.19	1.52	0.19	1.52	0.19	1.59	0.18	1.55	0.18	1.40	0.17	1.54	0.16	1.44	0.16	1.37	0.19	1.50	0.18	1.55
	**TAG**	0.09	0.74	0.09	0.74	0.07	0.61	0.09	0.76	0.11	0.87	0.08	0.69	0.09	0.83	0.09	0.83	0.10	0.77	0.09	0.76
	**TGA**	0.09	0.74	0.09	0.74	0.10	0.80	0.08	0.68	0.09	0.72	0.09	0.77	0.08	0.72	0.09	0.80	0.09	0.73	0.08	0.68
**Thr**	**ACA**	1.56	1.23	1.54	1.22	1.60	1.25	1.57	1.23	1.57	1.25	1.53	1.21	1.55	1.22	1.54	1.22	1.50	1.20	1.57	1.24
	**ACC**	0.89	0.70	0.90	0.71	0.88	0.69	0.89	0.70	0.87	0.69	0.87	0.69	0.89	0.70	0.90	0.71	0.88	0.70	0.87	0.69
	**ACG**	0.56	0.45	0.52	0.41	0.53	0.41	0.52	0.41	0.52	0.41	0.52	0.41	0.54	0.42	0.54	0.43	0.51	0.41	0.54	0.42
	**ACT**	2.04	1.61	2.11	1.66	2.12	1.65	2.11	1.66	2.06	1.64	2.12	1.68	2.09	1.65	2.04	1.62	2.11	1.69	2.08	1.64
**Trp**	**TGG**	1.74	1.00	1.77	1.00	1.79	1.00	1.78	1.00	1.76	1.00	1.77	1.00	1.77	1.00	1.76	1.00	1.75	1.00	1.78	1.00
**Tyr**	**TAC**	0.71	0.41	0.72	0.41	0.69	0.38	0.72	0.39	0.66	0.38	0.69	0.37	0.71	0.38	0.71	0.39	0.71	0.40	0.71	0.38
	**TAT**	2.78	1.59	2.81	1.59	2.87	1.61	2.94	1.61	2.81	1.62	2.99	1.62	2.97	1.61	2.97	1.61	2.81	1.59	2.96	1.61
**Val**	**GTA**	1.98	1.46	1.95	1.45	2.01	1.46	1.94	1.44	1.94	1.42	1.92	1.43	1.90	1.40	1.93	1.45	2.01	1.46	1.93	1.43
	**GTC**	0.67	0.49	0.63	0.46	0.67	0.48	0.67	0.50	0.72	0.53	0.69	0.51	0.68	0.51	0.64	0.48	0.67	0.49	0.64	0.47
	**GTG**	0.84	0.62	0.83	0.62	0.81	0.58	0.80	0.60	0.83	0.61	0.80	0.59	0.82	0.61	0.79	0.59	0.84	0.61	0.81	0.60
	**GTT**	1.93	1.42	1.97	1.46	2.02	1.47	1.98	1.47	1.95	1.43	1.97	1.46	2.00	1.47	1.96	1.47	1.95	1.42	2.01	1.49
