# Supplementary material for: Plastid phylogenomics of Pleurothallidinae (Orchidaceae): Conservative plastomes, new variable markers, and comparative analyses of plastid, nuclear, and mitochondrial data
Source: PLoS One. 2021 Aug 27;16(8):e0256126. doi: 10.1371/journal.pone.0256126 (PMC8396723; doi:10.1371/journal.pone.0256126)
Supplement: S7 Table — D > 0 = purifying selection, and D < 0 = positive selection. Significant results (p ≤ 0.05) were highlighted in bold. *Tajima’s D test was not computed due to the lack of variable sites. (PDF) [file pone.0256126.s013.pdf]

CDS	*D*	*P* value	CDS	*D*	*P* value
accD	**-4.36**	**< 0.01**	***psbM***	-1.03	0.30
atpA	-1.49	0.13	***psbN***	-1.40	0.16
atpB	**-2.61**	**0.01**	***psbT***	**-3.55**	**< 0.01**
atpE	-0.89	0.37	***psbZ***	-0.67	0.50
atpF	-1.18	0.24	***rbcL***	**-2.32**	**0.02**
atpH	-0.96	0.34	***rpl2***	-1.01	0.31
atpI	-1.51	0.13	***rpl14***	-0.83	0.40
ccsA	-1.60	0.11	***rpl16***	-0.38	0.70
cemA	-1.28	0.20	***rpl20***	-1.41	0.16
clpP	-1.02	0.30	***rpl22***	**-2.03**	**0.04**
infA	-0.03	0.98	***rpl23***	-1.40	0.16
matK	-1.26	0.21	***rpl32***	**-1.96**	**0.05**
petA	-1.17	0.24	***rpl33***	**-1.95**	**0.05**
petB	**-3.49**	**< 0.01**	***rpl36***	-1.40	0.16
petD	-1.27	0.20	***rpoA***	-1.35	0.17
petG	-1.56	0.12	***rpoB***	-1.57	0.11
petL	-1.40	0.16	***rpoC1***	**-2.20**	**0.03**
petN	-0.69	0.49	***rpoC2***	-1.44	0.15
psaA	-1.58	0.11	***rps2***	-1.68	0.09
psaB	-1.28	0.20	***rps3***	-1.43	0.15
psaC	-1.84	0.06	***rps4***	-1.65	0.10
psaI	-0.63	0.53	***rps7****	-	-
psaJ	1.13	0.26	***rps8***	-1.65	0.10
psbA	-1.23	0.22	***rps11***	-1.50	0.13
psbB	**-1.91**	**0.05**	***rps12***	-1.11	0.26
psbC	-0.70	0.48	***rps14***	-1.01	0.31
psbD	-1.15	0.25	***rps15***	-1.80	0.07
psbE	-1.74	0.08	***rps16***	-1.38	0.70
psbF	-1.39	0.16	***rps18***	**-3.58**	**< 0.01**
psbH	-1.59	0.11	***rps19***	-1.39	0.16
psbI	0.01	0.99	***ycf1***	**-3.22**	**< 0.01**
psbJ	-1.40	0.16	***ycf2***	**-2.19**	**0.03**
psbK	-1.75	0.08	***ycf3***	-0.85	0.39
psbL	-1.11	0.26	***yfc4***	-1.80	0.07
