# Supplementary material for: Plastid phylogenomics of Pleurothallidinae (Orchidaceae): Conservative plastomes, new variable markers, and comparative analyses of plastid, nuclear, and mitochondrial data
Source: PLoS One. 2021 Aug 27;16(8):e0256126. doi: 10.1371/journal.pone.0256126 (PMC8396723; doi:10.1371/journal.pone.0256126)
Supplement: S8 Table — (PDF) [file pone.0256126.s014.pdf]

Plastome	SSRs count	SSRs by repeat unit size						SSRs by location			SSRs by region		
		Mono	Di	Tri	Tetra	Penta	Hexa	Exons	IGSs	Introns	IR	LSC	SSC
Acianthera recurva	116	33	10	58	9	6	0	34	64	18	22	87	7
Anathallis microphyta	135	59	7	57	8	1	3	44	68	23	13	98	25
Anathallis obovata	134	62	5	57	8	1	1	41	73	20	17	94	23
Dryadella lilliputiana	143	65	4	62	9	2	1	41	84	18	18	96	29
Masdevallia picturata	131	51	8	61	11	0	0	44	69	18	17	92	22
Myoxanthus exasperatus	122	46	8	57	7	3	1	38	70	14	19	86	17
Octomeria grandiflora	127	56	6	57	5	2	1	40	72	15	18	90	19
Pabstiella mirabilis	113	41	9	54	6	3	0	40	62	12	16	80	17
Stelis grandiflora	133	62	5	54	10	2	0	41	76	16	16	95	22
Stelis montserratii	136	63	8	54	9	2	0	41	80	15	18	98	20
**Total**	1,290	538	70	571	82	22	7	363	718	209	174	916	200
