# Supplementary material for: Plastid phylogenomics of Pleurothallidinae (Orchidaceae): Conservative plastomes, new variable markers, and comparative analyses of plastid, nuclear, and mitochondrial data
Source: PLoS One. 2021 Aug 27;16(8):e0256126. doi: 10.1371/journal.pone.0256126 (PMC8396723; doi:10.1371/journal.pone.0256126)
Supplement: S9 Table — SSR type in brackets with repetition frequency following. Loci amplified by the same primer separated by semicolons. Slanting bars (/) indicate polymorphism in repeat units. Tm = primer melting temperature. (PDF) [file pone.0256126.s015.pdf]

Primer name	Product size (bp)	Location	Region	SSR type	Primer sequence (5'−3')	Tm (ºC)	% GC
**Pleuro_SSR1**	341	*psbA-trnK* IGS	LSC	(T) × 10−17	F - TTGCAGAAGCGACCCCATAG<br>R - CGACTAGTTCCGGGTTCGAG	60.1<br>59.9	55.0<br>60.0
**Pleuro_SSR2**	422	*psbK-psbI* IGS	LSC	(A) × 10−15; (ATCTT) × 3−4	F - AGGGAAGGAGGACGATGATCT<br>R - GGATTACGCCCCGGATCATT	59.8<br>60.0	52.4<br>55.0
**Pleuro_SSR3**	442	*atpH-atpI* IGS	LSC	(TAC) × 3	F - CAAGCCCTACAGCCAATCCA<br>R - AGCCCACTGCATTATAGATGGA	60.0<br>59.0	55.0<br>45.0
**Pleuro_SSR4**	421	*atpI-rps2* IGS	LSC	(TTC) × 3	F - TGTTGGCCTACTTCTACACCTG<br>R - GCAATTTGTGAGGGCCGTTC	59.7<br>60.4	50.0<br>55.0
**Pleuro_SSR5**	365	*rps2-rpoC2* IGS	LSC	(T) × 10−16	F - GCCCTTATTGCTGCTGATGC<br>R - CTGGTAGAAGGTTCCGTCGG	60.0<br>59.8	55.0<br>60.0
**Pleuro_SSR6**	226	*rpoC1* intron	LSC	(TCC) × 3	F - TCATATTCGCTTGCGCTTGC<br>R - CCCCGGCTCTGACATGTATC	60.0<br>60.0	50.0<br>60.0
**Pleuro_SSR7**	296	*trnC-petN* IGS	LSC	(T) × 10−15	F - GGCTCTGAGTGCTGTTCCTT<br>R - TTAAAGCAGCCCAAGCGAGA	60.0<br>60.0	55.0<br>50.0
**Pleuro_SSR8**	457	*petN-psbM* IGS	LSC	(TTA) × 3	F - TATGGGGAAGGAGTGGGCTT<br>R - GAATTGCAAGCCCAACTGCT	60.3<br>59.7	55.0<br>50.0
**Pleuro_SSR9**	498	*trnE-trnT* IGS	LSC	(AGT) × 3−4 / (GTA) × 3	F - TGAGCTATCCCATACTCCCAT<br>R - CCACTGAGTTAAAAGGGCCCT	57.4<br>59.9	47.6<br>52.4
**Pleuro_SSR10**	110	*trnT-psbD* IGS	LSC	(A) × 10−15; (ATT) / (TTA) × 3	F - TCTTCGTTTTTCAGCGGGGA<br>R - ACGGGGAAGACTCCTCCTAA	59.9<br>59.3	50.0<br>55.0
**Pleuro_SSR11**	370	*psaA* CDS	LSC	(TTG) × 3	F - ACGGGAACTGCGAGCAAATA<br>R - ACATCTGAACTGGGCATGCA	60.0<br>60.0	50.0<br>50.0
**Pleuro_SSR12**	498	*psaA-ycf3* IGS	LSC	(T) × 10−15	F - TGCCAAAGCATTTCCCAGGA<br>R - GATTCGGAAATTGCGGAGGC	60.2<br>60.0	50.0<br>55.0
**Pleuro_SSR13**	134	*ycf3* intron I	LSC	(AAG) / (AAT) × 3	F - ATACGGCTCCTCCCTTCTGT<br>R - AACCTCTTGTGGGAAGGCTG	60.0<br>59.9	55.0<br>55.0
**Pleuro_SSR14**	349	*trnT-trnL* IGS	LSC	(A) × 10−15	F - GGGCGGGGATATAAGAGCAA<br>R - TGCCTTTCCTCCTCCTTCAT	59.3<br>58.3	55.0<br>50.0
**Pleuro_SSR15**	361	*trnT-trnL* IGS	LSC	(AGG) × 3; (AT) × 5−6	F - AGAATGAATATCGACCGTTCCACT<br>R - AGCGTCTACCAATTTCGCCA	59.9<br>60.0	41.7<br>50.0
**Pleuro_SSR16**	419	*trnL-trnF* IGS	LSC	(T) × 10−13	F - ATCGTGAGGGTTCAAGTCCC<br>R - TTCCCGTGCATCATCCTAGC	59.4<br>59.9	55.0<br>55.0
**Pleuro_SSR17**	476	*ndhC-trnV* IGS	LSC	(A) × 11−15	F - TGTGATGTGTAGACATAGCATGC<br>R - CGGTTCGAGTCCGTATAGCC	58.9<br>60.0	43.5<br>60.0
**Pleuro_SSR18**	517	*atpB-rbcL* IGS	LSC	(TA) × 4−8	F - TACTGTCAAGAGAGGGGGCC<br>R - GCGCAACCCAATCTTCGTTT	60.6<br>60.0	60.0<br>50.0

**Pleuro_SSR19**	430	*accD* CDS	LSC	(TAT) × 3−4	F - ACCGGTGGGAGTGAAGAAAC<br>R - AACGAAGATAACTATCAATGCAACT	59.9<br>57.2	55.0<br>32.0
**Pleuro_SSR20**	454	*accD-psaI* IGS	LSC	(A) × 10−19	F - TGGAAGTATATCACTAGCTTCAGTT<br>R - TGAAAAACGGGGGTTTTCGC	57.3<br>59.9	36.0<br>50.0
**Pleuro_SSR21**	450	*accD-psaI* IGS	LSC	(AGA) × 3	F - ACCCCCGTTTTTCACTAGGA<br>R - AAGAAGCCATTGCGATTGCC	58.6<br>59.8	50.0<br>50.0
**Pleuro_SSR22**	242	*ycf4-cemA* IGS	LSC	(TAA) × 3−5 / (AAT) × 3	F - CTTGCGCGTACCCATTGAAG<br>R - AAGAATGAAGCTGGACGCCA	59.9<br>60.0	55.0<br>50.0
**Pleuro_SSR23**	500	*petA-psbJ* IGS	LSC	(T) × 10−19	F - GTTTCGGCTGAAAAGCGGAT<br>R - AAGAACTCAGCGGGGTAAGG	59.5<br>59.4	50.0<br>55.0
**Pleuro_SSR24**	388	*petL-trnW* IGS	LSC	(CCT) × 3	F - CGCGTCAATTGCCAATTCCT<br>R - GGGTCTCCAAAACCCGATGT	59.8<br>60.0	50.0<br>55.0
**Pleuro_SSR25**	431	*trnP-psaJ* IGS	LSC	(AAG) × 3	F - AGCTGCGCTACATCCCTTTT<br>R - CACAGGTGCCGTTGAGAGAT	60.0<br>60.0	50.0<br>55.0
**Pleuro_SSR26**	327	*psaJ-rpl33* IGS	LSC	(A) × 11−18	F - ATCTCTCAACGGCACCTGTG<br>R - CGCGCTCCTAGACTGAATCC	60.0<br>60.3	55.0<br>60.0
**Pleuro_SSR27**	461	*rps18-rpl20* IGS	LSC	(AAT) × 10; (CTA) × 3	F - TTTCGTAGGCGTTTACCCCC<br>R - GGAGAATGGACTCCGGGAAG	60.0<br>59.5	55.0<br>60.0
**Pleuro_SSR28**	500	*rpl20-rps12* IGS	LSC	(T) × 10−11	F - CACCGGAGCCTCTTCTTTCA<br>R - TGCGACTCGTTCAGTTCAGA	59.7<br>59.3	55.0<br>50.0
**Pleuro_SSR29**	476	*clpP* CDS; intron I	LSC	(ATAA) × 3; (T) × 10−13	F - GGTATTACCCATCCGCCTGG<br>R - TTCGGACAAATCTTCCCCCG	60.0<br>60.0	60.0<br>55.0
**Pleuro_SSR30**	413	*clpP* intron I	LSC	(T) × 10−12	F - CGGGGGAAGATTTGTCCGAA<br>R - ATCCAGGCTCCGTTCAGAGA	60.0<br>60.3	55.0<br>55.0
**Pleuro_SSR31**	433	*clpP-psbB* IGS	LSC	(TAT) × 3; (AT) × 6−23 / (TA) × 5−17	F - TGGAAACGTAACAATGGTTTTATTG<br>R - GACTGATCCGATCGATCCCG	57.1<br>59.8	32.0<br>60.0
**Pleuro_SSR32**	585	*psbB-psbT* IGS	LSC	(T) × 11−16	F - GCGCCAGAACCTTGTTCAGA<br>R - GCTTCCACACCTATTCATTTTGGA	60.9<br>59.5	55.0<br>41.7
**Pleuro_SSR33**	206	*petD* intron	LSC	(T) × 11−19	F - ACAGGCTCCGTAAGATCCCT<br>R - GATCAGGGTCGATGCAGAGG	60.0<br>60.0	55.0<br>60.0
**Pleuro_SSR34**	456	*rpoA* CDS	LSC	(TCT) × 3−4	F - GTGCTTCTGTAGAGTGCCCA<br>R - TCTATGCCTGTTCGAAATGCG	59.7<br>59.1	55.0<br>47.6
**Pleuro_SSR35**	308	*rpl36-infA* IGS	LSC	(T) × 10−19	F - ATAATTCGACCCCGCCTACG<br>R - TCCGGCGCAGTTTTATACGT	59.7<br>60.1	55.0<br>50.0
**Pleuro_SSR36**	294	*rpl36-infA* IGS	LSC	(T) × 10−17	F - TGTCCCTACCCATGACGAAC<br>R - TTTTGGTGCAATTGCTCGGG	59.1<br>60.0	55.0<br>50.0
**Pleuro_SSR37**	422	*rpl14-rpl16* IGS	LSC	(CTT) × 3−4 / (TCT) × 3	F - TGAGCTCCACTGTTATCCGC<br>R - TATGCACGTCGTGGTGGAAA	59.8<br>60.0	55.0<br>50.0

**Pleuro_SSR38**	347	*rpl16* intron	LSC	(CTT) × 3−4 / (TCT) × 3	F - GCTTAGGCCTGAACTCTGCA<br>R - GGCAGTGTTATCAAGCATCAACA	60.0<br>59.8	55.0<br>43.5
**Pleuro_SSR39**	481	*ycf2* CDS	IR	(CTT) × 3	F - TGCGTTGAGAAAGGGCAGAT<br>R - ACTCACTAGAGGCTCGGGAA	60.0<br>59.7	50.0<br>55.0
**Pleuro_SSR40**	293	*ycf2* CDS	IR	(TGA) × 3	F - AACCCTTTTCGCTCCGCTTA<br>R - TGGGTCTATTTTCGGCGTCA	60.0<br>59.4	50.0<br>50.0
**Pleuro_SSR41**	410	*ycf2* CDS	IR	(CTT) × 3	F - TGACGCCGAAAATAGACCCA<br>R - TGCTACAAGATCTCGCGCAT	59.4<br>59.9	50.0<br>50.0
**Pleuro_SSR42**	353	*ycf2* CDS	IR	(GAA) × 3	F - ATGCGCGAGATCTTGTAGCA<br>R - CGATCCGGCAGAACAACTCA	59.9<br>60.4	50.0<br>55.0
**Pleuro_SSR43**	500	*rps12-trnV* IGS	IR	(CTT) × 3	F - CCTCATACGGCTCCTCGTTC<br>R - CCCCTCGCTCGATGAGAAAA	60.0<br>59.8	60.0<br>55.0
**Pleuro_SSR44**	375	*rps12-trnV* IGS	IR	(TCC) × 3	F - TTCTCATCGAGCGAGGGGTA<br>R - CCCTTACGCAATCGATCGGA	60.1<br>60.0	55.0<br>55.0
**Pleuro_SSR45**	432	*rps12-trnV* IGS	IR	(T) × 10−13	F – TTGGCGCAAGAATAAGGGGT<br>R - TGACTTCCACCACGTCAAGG	60.0<br>59.9	50.0<br>55.0
**Pleuro_SSR46**	281	*ycf1* CDS	IR	(CAA) × 3	F – ACCAAGTTCCACGTTAGCCA<br>R - TAGTGACAATAGGCGACGGC	59.5<br>59.9	50.0<br>55.0
**Pleuro_SSR47**	398	*ndhF* CDS	SSC	(CAA) × 3	F – GGTCGTGTGAACCAAAAGCC<br>R - CGTCGTATGTGGGCTTTCCT	60.0<br>60.1	55.0<br>55.0
**Pleuro_SSR48**	290	*rpl32-ccsA* IGS	SSC	(TTA) × 3−6 / (ATT) × 3	F – ACCCCGACTGAGAACGAAAC<br>R - TGCTTCCTAAGAGCAGCGTG	60.0<br>60.4	55.0<br>55.0
**Pleuro_SSR49**	260	*ndhE-ndhG* IGS	SSC	(A) × 10−14	F – TGCTTCGGCTTGTGATCAGT<br>R - AGGTGCAATTACTATGGCTCGT	60.0<br>59.8	50.0<br>45.5
**Pleuro_SSR50**	405	*ycf1* CDS	SSC	(T) × 10−11	F – TGACTCATAAATGAATTCGCTGCA<br>R - ATCTTCCCTGTCCCAAGCAT	59.4<br>58.7	37.5<br>50.0
**Pleuro_SSR51**	405	*ycf1* CDS	SSC	(A) × 10	F – AACGGACGATTCGCGAAGAA<br>R - GTCTGGGGAAAGGAAGCGAA	60.4<br>60.0	50.0<br>55.0
**Pleuro_SSR52**	422	*ycf1* CDS	SSC	(TTA) × 3	F – CCTCATCCCACTCAGTAATTTGGA<br>R - AGGATCTATGCGCGCTCAAA	60.1<br>59.9	45.8<br>50.0
**Pleuro_SSR53**	464	*ycf1* CDS	SSC	(T) × 12−14; (TCA) × 3	F – CCTCTTCTCCCAATTCTCCCA<br>R - TGAGAGATCCCCTAGTCTGGA	58.8<br>58.5	52.4<br>52.4
**Pleuro_SSR54**	300	*ycf1* CDS	SSC	(T) × 10−11; (A) × 12	F – GGATTCGACTTCTCAATTCATTGT<br>R - CTTTTCTTTTCGATTATAAACGATGGA	57.6<br>57.0	37.5<br>29.6
