# Supplementary material for: Plastid phylogenomics of Pleurothallidinae (Orchidaceae): Conservative plastomes, new variable markers, and comparative analyses of plastid, nuclear, and mitochondrial data
Source: PLoS One. 2021 Aug 27;16(8):e0256126. doi: 10.1371/journal.pone.0256126 (PMC8396723; doi:10.1371/journal.pone.0256126)
Supplement: S10 Table — In total, 104 sequences were analyzed, comprising intergenic spacers (IGS), introns, and popular molecular markers for Orchidaceae such as the trnH-psbA IGS, the matK CDS, and the 3’ portion of ycf1 CDSs. IGS = inter-genic spacer, CDS = protein-coding sequence. (PDF) [file pone.0256126.s016.pdf]

**Sequence name**	**SV (%)**	**Location**	**Region**	**Length (bp)**	**GC (%)**
psbI-trnS^{GCU}	41.67	IGS	LSC	158	22.8
ndhF-rpl32	41.19	IGS	SSC	1149	18.5
psbB-psbT	38.76	IGS	LSC	610	19.5
trnS^{GCU}***-trnG***^{UCC}	36.19	IGS	LSC	1334	26.5
rpl32-trnL^{UAG}	33.88	IGS	SSC	996	23.8
trnR^{UCU}***-atpA***	31.94	IGS	LSC	224	23.4
trnW^{CCA}***-trnP***^{UGG}	30.61	IGS	LSC	200	30.8
petN-psbM	29.78	IGS	LSC	1034	25.6
rpl16-rps3	29.68	IGS	LSC	192	22.2
psbK-psbI	29.63	IGS	LSC	611	25.1
atpB-rbcL	29.04	IGS	LSC	1319	23.9
clpP-psbB	28.38	IGS	LSC	1111	24.0
rps8-rpl14	28.13	IGS	LSC	265	21.1
psbM-trnD^{GUC}	26.95	IGS	LSC	1370	30.9
atpF-atpH	26.58	IGS	LSC	183	26.3
rps15-ycf1	26.53	IGS	SSC	459	24.0
trnG^{UCC}***-trnR***^{UCU}	26.50	IGS	LSC	166	24.7
trnP^{UGG}***-psaJ***	26.29	IGS	LSC	497	23.9
atpH-atpI	26.27	IGS	LSC	809	30.0
trnT^{UGU}***-trnL***^{UAA}	25.50	IGS	LSC	734	26.9
trnE^{UUC}***-trnT***^{GGU}	25.32	IGS	LSC	854	24.7
accD-psaI	24.54	IGS	LSC	887	27.2
ndhC-trnV^{UAC}	24.51	IGS	LSC	830	25.4
trnG^{GCC}***-trnfM***^{CAU}	24.34	IGS	LSC	215	32.6
trnF^{GAA}***-ndhJ***	24.00	IGS	LSC	191	27.2
trnK^{UUU}***-matK***	23.95	intron	LSC	311	29.0
ycf4-cemA	22.84	IGS	LSC	832	31.1
trnD^{GUC}***-trnY***^{GUA}	22.53	IGS	LSC	403	29.6
rps16-trnQ^{UUG}	22.39	IGS	LSC	479	26.8
trnK^{UUU}***-rps16***	22.33	IGS	LSC	605	27.6
psaC-ndhE	22.03	IGS	SSC	610	21.7
psbA-trnK^{UUU}	21.93	IGS	LSC	275	28.0
rpl33-rps18	21.47	IGS	LSC	214	27.5
matK- trnK^{UUU}	21.36	intron	LSC	1305	28.5
petA-psbJ	21.35	IGS	LSC	1163	28.8
rpl16	21.23	intron	LSC	1484	27.9
ccsA-ndhD	21.09	IGS	SSC	297	23.8
psbC-trnS^{UGA}	21.01	IGS	LSC	150	35.3
trnT^{GGU}***-psbD***	20.77	IGS	LSC	1006	33.7
ndhE-ndhG	20.65	IGS	SSC	161	22.5
psbE-petL	20.39	IGS	LSC	1320	27.6
psbZ-trnG^{GCC}	20.31	IGS	LSC	328	31.3
trnL^{UAA}***-trnF***^{GAA}	20.24	IGS	LSC	441	30.9
***ycf1* (3' portion)**	20.05	CDS	SSC	4541	27.5
trnH^{GUG}***-psbA***	19.94	IGS	IR/LSC	820	34.8
psaJ-rpl33	19.90	IGS	LSC	710	25.8
trnQ^{UUG}***-psbK***	19.85	IGS	LSC	390	23.4
ndhA	19.59	intron	SSC	1181	30.4
trnL^{UAG}***-ccsA***	19.49	IGS	SSC	160	18.7
atpI-rps2	19.35	IGS	LSC	309	25.6
psbH-petB	19.35	IGS	LSC	226	26.3
rps18-rpl20	19.25	IGS	LSC	274	30.9
petL-petG	19.17	IGS	LSC	192	29.8
rpoB-trnC^{GCA}	18.97	IGS	LSC	1537	30.4
ndhG-ndhI	18.92	IGS	SSC	246	27.0
ycf3-trnS^{GGA}	18.83	IGS	LSC	620	31.5
rpl22-rps19	18.60	IGS	IR	270	30.0
trnL^{UAA}	18.56	intron	LSC	827	30.2

rbcL-accD	18.33	IGS	LSC	922	30.6
trnC^{GCA}***-petN***	17.81	IGS	LSC	875	33.6
rpoC2-rpoC1	17.51	IGS	LSC	205	36.9
trnfM^{CAU}***-rps14***	17.50	IGS	LSC	172	35.1
rps4-trnT^{UGU}	17.42	IGS	LSC	365	27.8
psaA-ycf3	17.35	IGS	LSC	668	29.8
trnS^{GGA}***-rps4***	17.26	IGS	LSC	400	28.6
psaI-ycf4	17.26	IGS	LSC	435	31.3
petD-rpoA	16.67	IGS	LSC	191	29.7
rps2-rpoC2	16.67	IGS	LSC	238	31.5
clpP **II**	16.57	intron	LSC	1068	30.7
rps16	16.20	intron	LSC	983	31.7
rps11-rpl36	15.85	IGS	LSC	197	25.5
atpF	15.70	intron	LSC	999	30.7
petB-petD	13.89	IGS	LSC	197	31.3
clpP **I**	12.88	intron	LSC	716	30.8
petD	12.81	intron	LSC	949	33.1
trnM^{CAU}***-atpE***	12.70	IGS	LSC	198	29.4
rpl20-rps12	12.45	IGS	LSC	838	34.9
cemA-petA	12.14	IGS	LSC	255	30.3
trnV^{UAC}***-trnM***^{CAU}	11.30	IGS	LSC	178	32.4
trnG^{UCC}	10.68	intron	LSC	709	31.4
matK	10.58	CDS	LSC	1542	32.2
trnS^{UGA}***-psbZ***	10.57	IGS	LSC	233	33.1
petB	10.47	intron	LSC	744	34.1
rpoC1	10.30	intron	LSC	791	36.3
trnR^{ACG}***-trnN***^{GUU}	09.68	IGS	IR	585	42.2
ycf3 **II**	09.18	intron	LSC	734	36.0
ycf3 **I**	08.66	intron	LSC	814	32.8
ndhD-psaC	08.21	IGS	SSC	152	34.5
trnL^{CAA}***-ndhB***	07.20	IGS	IR	588	37.1
trnV^{UAC}	06.84	intron	LSC	589	39.6
ycf2-trnI^{CAU}	06.25	IGS	IR	999	42.7
rps12-trnV^{GAC}	06.23	IGS	IR	2005	39.7
rpl23-trnI^{CAU}	05.16	IGS	IR	165	34.6
rrn5-trnR^{ACG}	05.00	IGS	IR	247	41.3
rrn16-trnI^{GAU}	04.76	IGS	IR	294	50.6
trnN^{GUU}***-ycf1***	04.04	IGS	IR	357	38.1
rrn4.5-rrn5	03.70	IGS	IR	217	46.3
ndhB	03.07	intron	IR	705	39.3
ndhB-rps7	02.52	IGS	IR	341	35.4
rps12	02.01	intron	IR	547	39.7
trnA^{UGC}	01.99	intron	IR	809	50.9
trnI^{GAU}	01.71	intron	IR	939	49.2
trnV^{GAC}***-rrn16***	01.71	IGS	IR	243	45.9
rpl2	01.20	intron	IR	665	40.6
