# Supplementary material for: Plastid phylogenomics of Pleurothallidinae (Orchidaceae): Conservative plastomes, new variable markers, and comparative analyses of plastid, nuclear, and mitochondrial data
Source: PLoS One. 2021 Aug 27;16(8):e0256126. doi: 10.1371/journal.pone.0256126 (PMC8396723; doi:10.1371/journal.pone.0256126)
Supplement: S11 Table — SV = sequence variability, Tm = primer melting temperature. *Primers previously published but with mismatches in Pleurothallidinae. For these cases, subtribe-specific primers for the same regions are provided. (PDF) [file pone.0256126.s017.pdf]

| Sequence | SV | Region | Primer sequence (5’−3’) | Tm (ºC) | %GC | Product size (bp) |
|---|---|---|---|---|---|---|
| ***ndhF-rpl32*** | 41.19 | SSC | ndhF* – CCAATATCCCTTYYTTTTCCAA [79]<br>rpl32R* – CCAATATCCCTTYYTTTTCCAA [79] | 60.0<br>60.0 | 52.4<br>50.0 | - |
| | | | Pleuro_ndhF-F – AGGAAAGCCCACATACGACG<br>Pleuro_rpl32-R – ACTTTTTGACTGTCCGGTGGA | 60.1<br>59.8 | 55.0<br>47.6 | 1068 |
| ***petN-psbM*** | 29.78 | LSC | Pleuro_petN-F – TCTCGCTTGGGCTGCTTTAA<br>Pleuro_psbM-R – GCGCTTATTGCTACTACGCTG | 60.0<br>59.8 | 50.0<br>52.4 | 973 |
| ***psbB-psbT*** | 38.76 | LSC | Pleuro_psbB-F – TGCTACGTTTGCTTTGCTCT<br>Pleuro_psbT-R – GGGGGCTCATTACTTCAATGGA | 58.4<br>60.1 | 45.0<br>50.0 | 800 |
| ***psbI-trnS$^{GCU}$*** | 41.67 | LSC | Pleuro_psbI-F – AATGATCCGGGGCGTAATCC<br>Pleuro_trnS(GCU)-R – ATGGGGAGAGATGGCTGAGT | 60.0<br>60.0 | 55.0<br>55.0 | 266 |
| ***psbK-psbI*** | 29.63 | LSC | psbK – TTAGCCTTTGTTTGGCAAG [80]<br>psbI* – AGAGTTTGAGAGTAAGCAT [80] | 54.1<br>50.7 | 42.1<br>36.8 | - |
| | | | Pleuro_psbI-R – GGATTACGCCCCGGATCATT | 60.0 | 55.0 | 699 |
| ***rpl16-rps3*** | 29.68 | LSC | Pleuro_rpl16-F – AACGAGTCACACACTGAGCA<br>Pleuro_rps3-R – TGCACGTGTCGAATGGATCA | 59.5<br>60.0 | 50.0<br>50.0 | 337 |
| ***rpl32-trnL$^{UAG}$*** | 33.88 | SSC | rpl32-F – CAGTTCCAAAAAAACGTACTTC [79]<br>trnL_UAG – CTGCTTCCTAAGAGCAGCGT [79] | 54.5<br>60.1 | 36.4<br>55.0 | - |
| ***trnR$^{UCU}$-atpA*** | 31.94 | LSC | Pleuro_trnR(UCU)-F – AATGAAGGGCGTCCATTGTCT<br>Pleuro_atpA-R – GCTGGAATCAGGCCTGCTAT | 60.0<br>59.9 | 47.6<br>55.0 | 700 |
| ***trnS$^{GCU}$-trnG$^{UCC}$*** | 36.19 | LSC | trnS* – AGATAGGGATTCGAACCCTCG [79]<br>trnG* – GTAGCGGGAATCGAACCCGCATC [79] | 58.8<br>65.8 | 52.4<br>60.9 | - |
| | | | Pleuro_trnS(GCU)-F – ACGCTTTAGTCCACTCAGCC<br>Pleuro_trnG(UCC)-R – AGCCGAGGGTTCTAGTAAACC | 60.0<br>59.2 | 55.0<br>52.4 | 1336 |
| ***trnW$^{CCA}$-trnP$^{UGG}$*** | 30.61 | LSC | Pleuro_trnW(CCA)-F – ATTTGAACCTACGACATCGGGT<br>Pleuro_trnP(UGG-R – GCTTGGTAGCGCGTTTGTTT | 59.8<br>60.3 | 45.5<br>50.0 | 300 |
