# Supplementary material for: Plastid phylogenomics of Pleurothallidinae (Orchidaceae): Conservative plastomes, new variable markers, and comparative analyses of plastid, nuclear, and mitochondrial data
Source: PLoS One. 2021 Aug 27;16(8):e0256126. doi: 10.1371/journal.pone.0256126 (PMC8396723; doi:10.1371/journal.pone.0256126)
Supplement: Attachment — Submitted filename: Response to Reviewers.docx [file pone.0256126.s021.docx]

We’d like to thank the Reviewers for the great contributions to our manuscript entitled “Phylogenomic study in Pleurothallidinae (Orchidaceae): conservative plastomes, new variable markers, and comparative analyses among plastid, nuclear, and mitochondrial data”. The corrections requested were relevant and improved our text, which will lead to a better publication in PLOS One.

In short, we revised the manuscript to meet PLOS One requirements. We also considered all Reviewers’ contributions. Most figures and tables were shifted to Supplementary Material, and the entire text was carefully revised by all co-authors, especially by the author who is a native English speaker. Responses to all considerations are provided below. We also changed the title to “Plastid phylogenomics of Pleurothallidinae (Orchidaceae): conservative plastomes, new variable markers, and comparative analyses of plastid, nuclear, and mitochondrial data”.

Reviewer #1:

1. There is too much figures and tables in the body part of the manuscript, and some figure and table can shift to supplementary section, such as Table 4, Table 5, Table 6, Table 7, Table 8. And Table 1 and Table 2 can combine in one table.

**R.:** The revised manuscript now contains only four figures and three tables. Former Figs 1 and 2 and Tables 1, 3, 4, 5, 6, and 7 moved to Supplementary Material. Former Figs 6 and 7 split into Fig 4 and S5 and S6 Figs.

2. Furthermore, Tajima’s D test is mainly used in population genetics, the result of positive selection in this study might contain false positives.

**R.:** Tajima’s D test computes the difference between the average number of nucleotide differences and the number of segregating sites, which is expected to not exist in neutrality (the evolution rate is the same as the mutation rate), or, in other words, in the absence of selection. Indeed, the test is widely used in population genetics, but since it only needs DNA polymorphism to assess neutrality we do not think it is inappropriate for use on our data. Besides, most of the CDSs under selection according to our results were also reported in several other phylogenomic studies to be under selection in Orchidaceae. It is true that there might be false positives, but this is true in all cases in which it is used. Our conclusion in this case is simply that these regions test positive for selection, and because we do not use this result in any additional calculations or conclusions we maintain that our use is appropriate and correctly interpreted.

3. Also, there are minor errors in the paper:

L121 Is “CLC Genomics” indicate “CLC Genomics Workbench” ?

**R.:** Yes, and all mentioned in the text were corrected to CLC Genomics Workbench.

4. L212-L213 The classification of the bootstrap is unreasonable.

**R.:** Bootstrap percentages are no longer classified into “weak, moderate or strong”, but the 95% limit used instead as proposed by Feselstein. Citation also substituted.

5. L295 “Zhitao et al.” should be “Niu et al.”. L577-L579 the citation of Reference 27 is incorrect.

**R.:** Citation corrected.

6. Fig. 1, the plastome structure of the eight species are quite similar, the plastome map of one species is enough.

**R.:** Fig. 1 shifted to Supplementary Material.

7. Fig. 6, the tree topology of the four datasets is identical, one is enough.

**R.:** Actually, the Epidendreae trees diverged in the position of *Masdevallia picturata*, and all Pleurothallidinae trees diverged in some way, so we thought it was important to show all topologies. Fig 4 (former Fig 6) now presents the Epidendreae plastome and hotspot trees to show (1) that the variation in hotspots represents well the whole plastomes and (2) the divergence in *M. picturata* positioning and also the Pleurothallidinae combined tree. All other trees moved to Supplementary Material.

Reviewer #2:

1. I do not recommend the combined analysis based on plastome, nuclear ribosomal DNA operon and mitochondrial DNA (Fig. 7). Plastome and nuclear ribosomal DNA operon is subject to gene conversion, making it effectively uniparentally inherited (although there is no guarantee that the same parental copy will be kept in nuclear ribosomal DNA operon), whereas mitochondrial DNA could exhibit two alleles, making interpretation of results sometimes challenging. In addition, the incongruences have been shown between plastome and nuclear ribosomal DNA operon, e.g., Dryadella. I recommend the combined analysis should be based on the incongruence length difference (ILD) test.

**R.:** Plastomes and mitochondrial genomes are mostly maternally inherited (in most cases, as in Pleurothallidinae), and thus they do not have more than one allele per individual (except for heteroplasmy, which is not the case here). Plastome sequences are not subject to gene conversions, so this reviewer is incorrect in this assertion. These genes are present in single non-recombining molecule (one chromosome). Our molecular data are perfectly combinable, and the incongruence found is presented and discussed, although the cause of disagreement is beyond the scope of this paper. If we tried to hide the incongruence or did not mention it, then there would be a problem. Plus, many other studies combine nuclear and plastid DNA to obtain more robust phylogenetic hypotheses despite gene tree incongruence. The ILD is not necessary when incongruence is clear and strongly supported.

2. I would like to suggest put the Figs. 2,3, 5,6, into Supporting Information, the same as Tables 1, 3, 5, 6, 7, 8, 9. It is difficult to read in body, so many figures and tables. The writing should be more focus on the topic or key, rather than everything moved out.

**R.:** This issue was also indicated by Reviewer #1. However, we do not agree with the suggestion that the writing was not focused or that everything was moved out to the figures and tables. The figures and tables provided only illustrated what was discussed in the text, and that is the reason why we agree in moving most of the figures and tables to the Supplementary Material.

3. Further the writing can be improved. Many sentences are unclear and would benefit from being rephrased. I marked several sentences that need to be rephrased in the abstract, but stopped doing so for the rest of the manuscript. e.g., Line 26, please check the “first”, the plasomes of Anathallis obovata and Masdevallia picturata have been reported in previous study. The research background should be stated.

**R.:** We did not receive any tracked-changed text, so we were not sure of what sentences should be rephrased and why. Nevertheless, we revised the entire manuscript carefully, including by the native English speaker author. We also provided the research background for the plastome sequences as required.
